# MgO Fertilizer Sole and Combined with Organic and Inorganic Fertilizers: Effect on Soil Chemical Properties, Turmeric Performance, and Quality in a Tropical Alfisol

**DOI:** 10.1155/2019/8140276

**Published:** 2019-06-20

**Authors:** Aruna Olasekan Adekiya, Elizabeth Temitope Alori, Christopher Muyiwa Aboyeji, Oluwagbenga Dunsin, Kehinde Abodunde Adegbite, Charity O. Aremu, Olasunkanmi Bamiro, Wutem Sunny Ejue, Faith Oluwatobi Okunlola, Omowumi Oluwanifemi Adesola

**Affiliations:** College of Agricultural Sciences, Landmark University, P. M. B. 1001, Omu-Aran, Kwara State, Nigeria

## Abstract

For soil fertility maintenance, secondary nutrient such as Magnesium (Mg) is always being neglected. However, its role is critical in the growth, yield, and quality of crops. Therefore, two field experiments were initiated in 2017 and 2018 to evaluate the response of soil chemical properties, performance, and qualities of turmeric (*Curcuma longa* L.) to Mg fertilizer alone and in combination with poultry manure (PM) and NPK 15-15-15 fertilizer (NPK). The treatments applied were the following: (i) PM alone at 8 t ha^−1^, (ii) NPK alone at 200 kg ha^−1^, (iii) Mg fertilizer alone (in form of MgO) at 20 kg ha^−1^, (iv) PM at 8 t ha^−1^ with MgO at 20 kg ha^−1^ (PM + Mg), (v) NPK at 200 kg ha^−1^ with MgO at 20kgha^−1^ (NPK + Mg), and (vi) control (no amendment of any kind). PM, NPK, and Mg fertilizers alone or NPK + Mg and PM + Mg increased soil chemical properties, performance (plant height, number of leaves, number of tillers, number of rhizomes, and fresh rhizome weight), and minerals and vitamins C and A contents of turmeric rhizome compared with the control. By averaging 2017 and 2018, Mg fertilizer alone increased the yield of turmeric by 10.8% compared with the control. For this experiment, NPK + Mg increased growth and yield of turmeric compared with other treatments. Also, averaging 2017 and 2018, NPK + Mg increased rhizome yield of turmeric by 13.6% and 10.6% compared with PM + Mg and NPK alone, respectively. Similarly, PM + Mg significantly improved mineral and vitamins contents compared with other treatments. Therefore, for those that desire turmeric rhizome for its nutritive value, PM + Mg is recommended. For those that want quantity, NPK + Mg is recommended.

## 1. Introduction

Turmeric (*Curcuma longa* L.) is a spice crop that belongs to the family Zingiberaceae. There have been increases in the demand for turmeric over the last decade because of its medicinal values. Turmeric rhizomes are endowed with a yellowish compound known as curcumin and volatile oils which are of great medicinal value to mankind. Turmeric is rich in minerals (Fe, Ca, Mg, and P) and vitamin A [1]. Various supplements and drinks have also been reported to be produced from turmeric.

Turmeric is a crop that can exhaust soil fertility quickly. Therefore, due to its long gestation period (about 8 months) and high productivity it requires high fertilizer input [2]. In the tropics, to maintain productivity for a viable crop production maintenance of soil fertility with addition of fertilizers is inevitable.

Maintaining soil fertility has been based on adding different manures and macronutrients from chemical fertilizers [3]. N, P, and K either individually or in combination are considered to be the main nutrient elements to be responsible for the performance and quality of crops [4]. Secondary nutrients such as Magnesium (Mg) had always been neglected. However, its singular role in the growth, yield, and quality of crops is germane [4]. Addition of secondary nutrients such as Mg with either organic or inorganic fertilizer would complement their uses and improve soil chemical properties and crop yield.

Mg is involved in many physiological and biochemical processes in plants. It functions as the central atom of the chlorophyll molecule in the light absorbing complex of chloroplasts and its contribution to photosynthetic fixation of CO_2_ [5, 6]. It is required to activate a number of enzymatic systems in plant tissues such as carbohydrate metabolism, the citric acid cycle in cell respiration, and oil synthesis. It also performs structural roles in plants linking together the subunits of ribosomes [7]. For improved human nutrition and food quality, Mg addition to daily food intake is now becoming an important issue globally [8].

Turmeric has been reported to respond well to NPK 15-15-15 fertilizer [9] and poultry manure (PM) [10]. For example, an experiment with four turmeric varieties with organic and inorganic fertilizers [11] reported higher yields at 4884 kgha^−1^ from farmyard manure (organic) compared with 4623 kgha^−1^ recorded with NPK fertilizer (inorganic) manure. Rhizome yield of turmeric was also reported to increase with increasing concentration of MgSO_4_ applied as foliar a spray [12]. Crops react in different ways to different chemical or organic fertilizers. Therefore appropriate fertilizer combinations could influence soil chemical properties, crop yield, and quality.

Response of sole Mg fertilizer in combination with organic and inorganic fertilizer to crops especially turmeric is scarce in the literature. Therefore, this study was to determine the effect of Mg fertilizer alone and in combination with organic (PM) and inorganic (NPK 15-15-15) fertilizers on soil chemical properties, growth, rhizome yield, and quality. In this experiment, we hypothesized that soil chemical properties and turmeric growth and yield will react differently to sole NPK fertilizer, PM, Mg fertilizer, and the combinations of Mg fertilizer with either NPK fertilizer or PM. Experiments were conducted to validate these working hypotheses to determine which combinations of Mg fertilizer would have the greatest effect.

## 2. Materials and Methods

### 2.1. Site Description and Treatment

The studies were carried out at Owo, Ondo State, Nigeria, in 2017 and 2018. Owo, Nigeria, receives a total mean annual rainfall of about 1300 mm and mean air temperature of about 32°C. The site is located at latitude 5°12′N and longitude 5°35′E. The area has a diurnal rainfall, with first season starting in March to July with a short dry spell in August known as “August break,” followed by the second rainy season between September and November. The soil at Owo is an Alfisol or Luvisol. The site used for the experiment was just recovered from one year after a continuous cropping to yam (*Dioscorea rotundata*) and maize (*Zea mays*) for two years.

The treatments in 2017 and 2018 were the following: (i) application of PM alone at the rate of 8 t ha^−1^ based on field recommendation for turmeric production [10]; (ii) application of NPK 15-15-15 fertilizer alone at the rate of 200 kg ha^−1^ based on field recommendation for turmeric production [9]; (iii) application of Mg fertilizer alone (in form of MgO) at the rate of 20 kg ha^−1^; (iv) application of PM at 8 t ha^−1^ with MgO at 20 kg ha^−1^; (v) application of NPK fertilizer at 200 kg ha^−1^ with MgO at 20kgha^−1^; (vi) control (no amendment of any kind). The experimental design used was RCBD with three replications. Plots were 2.5 × 2.5 m with 0.5 m apart and 1 m space between blocks. The same site, layout, and treatments used in 2017 were repeated in 2018.

### 2.2. Incorporation of PM, Crop Establishment, and Applications of NPK 15-15-15 and MgO Fertilizers

The PM was composted two weeks to mineralize before application. Ploughing and harrowing (land preparation) were done in April in each year 2017 and 2018. The PM was applied to the required plots at three weeks prior to planting of turmeric rhizomes. At planting turmeric rhizomes were cut to about 50 g and treated with a fungicide (Benlate) to wade against seed and soil-borne pathogens before planting rhizomes using a hand trowel to a depth of about 6 cm at a spacing of 30 cm × 30 cm to give a plant population of 111,111 plants ha^−1^. NPK 15-15-15 fertilizer and MgO fertilizers were applied to the required plots 4 weeks after planting and after the first weeding. The fertilizers were applied 10 cm away from the turmeric plant by ring method. Weeding was subsequently done every month to keep the plots clean.

### 2.3. Turmeric Performance

Ten randomly selected turmeric plants were used for the determination of growth parameter (plant height, number of leaves, and tillers) after 150 days of turmeric growth when the turmeric would have developed full canopy formation. Plant height was taken by measuring from the ground level to the tallest leaf apex using a ruler. The numbers of leaves were counted. At harvesting (8 months after planting), individual plants were uprooted using hoe. The numbers of rhizomes were counted and the fresh rhizome weights of turmeric were recorded using a top loading balance.

### 2.4. Determination of Soil Properties

Before experimentation in 2017, soil samples (0–15 cm) were collected from 10 randomly selected locations within the experimental site and mixed together to represent a composite soil sample. The soil sample was later air-dried sieved using a 2 mm sieve and analyzed for particle size, organic C, pH, total N, available P, exchangeable K, Ca, Mg, and pH (water). At the end of each year, soil samples were also collected on each experimental plot and the chemical properties were analyzed. Particle size analysis was determined using the hydrometer method [13]. Soil organic carbon was determined by the procedure of Walkley and Black using the dichromate wet oxidation method [14]. Organic matter was calculated by multiplying C by 1.724. Total N was determined by the micro-Kjeldahl digestion method [15]. Available P was determined by Bray-1 extraction followed by molybdenum blue colorimetry [16]. Exchangeable K, Ca, and Mg were extracted using 1 M ammonium acetate. Thereafter K level was determined on a flame photometer, and Ca and Mg were determined by EDTA titration [17]. Soil pH was determined using a soil-water medium at a ratio of 1:2 with a digital electronic pH meter.

### 2.5. Chemical Analysis of PM and Turmeric Rhizome

Turmeric rhizomes each year were harvested and analyzed for minerals (Na, K, Mg, Ca, and Fe) and vitamins A and C. The minerals in the turmeric rhizome were analyzed using the method described by Association of Official Analytical Chemist [18]. Vitamin A was determined spectrophotometrically by using the hexane method while Vitamin C was extracted from the turmeric with oxalic acid and then titrated using 2, 6-dichlorophenol indophenol [19]. The PM was air dried and sieved and then analyzed for chemical properties (pH (water), ash, organic C, N, P, K, Ca, Mg, Na, Cu, Mn, S, and Zn) using the method recommended by [20].

### 2.6. Statistical Analysis

Data were subjected to analysis of variance (ANOVA) using SPSS 21, and means were separated using Duncan's multiple range test (DMRT) at p < 0.05 probability level.

## 3. Results

### 3.1. Initial Soil Fertility and Chemical Analysis of the Soil Amendments Used for the Experiment

Tables 1 and 2, respectively, show the initial soil physical and chemical properties of the site used before experimentation and the chemical analysis of the PM, NPK, and MgO fertilizers used for the study. The soil was sandy loam, acidic, and low in OM, N, P, Ca, and Mg but adequate in K [21]. The poultry manure has both macro- and microelements. NPK fertilizer has high values of N, P, and K while MgO has high Mg content compared with other amendments.

### 3.2. Response of Soil Chemical Properties

Although PM alone and PM with Mg fertilizer (PM + Mg) increased pH, OM, N, P, K, Ca, and Mg contents of the soil compared with the control, their application only resulted in significant differences (p < 0.05) for Mg (Table 3). NPK alone and NPK with Mg fertilizer (NPK + Mg) significantly increased N, P, K, and Mg concentrations of the soil compared with the control. Their application however did not significantly increase (p < 0.05) pH, OM, and Ca concentration compared with the control. NPK +Mg fertilizer increased soil Mg concentration significantly compared with NPK alone. Mg alone significantly increased N, P, K, Ca, and Mg concentrations of the soil compared with the control whereas the values of pH and OM were not significantly different. Compared with other treatments, NPK + Mg significantly (p < 0.05) increased N, P, and K concentrations of the soil. When compared with other treatments, PM + Mg significantly (p < 0.05) increased soil pH, OM, Ca, and Mg concentrations.

### 3.3. Response of Growth and Yield of Turmeric

Averaged over the two years, all amendments increased plant height, number of leaves, number of tillers, number of rhizomes, and turmeric rhizome yield compared with the control (Table 4). PM + Mg significantly increased growth and yield parameters except plant height compared to PM alone. Using the two-year average, PM + Mg increased turmeric yield by 7.8% compared with PM alone. NPK + Mg significantly increased growth and yield of turmeric compared to the other treatments. On average, NPK + Mg increased rhizome yield of turmeric by 13.6% and 10.6% compared with PM + Mg and NPK fertilizer alone, respectively. Similarly, Mg fertilizer alone increased the yield of turmeric by 10.8% compared with the control.

### 3.4. Response of Turmeric Mineral and Vitamins

All amendments significantly increased (p < 0.05) turmeric rhizomes' Na, K, Mg, Ca, Fe, and vitamins C and A compared with the control (Table 5). With the exception of K, PM + Mg significantly increased the mineral and vitamins in turmeric rhizomes compared with the other treatments. The values of Fe, Ca, and vitamin C for PM + Mg were not significantly different from that of PM alone. NPK + Mg had higher value of K compared with PM + Mg. Except for K, PM alone increased mineral and vitamins contents of turmeric rhizome compared with NPK fertilizer alone. Except for Mg, the Mg fertilizer alone had the least effect on mineral and vitamin content of turmeric compared to all other soil amendments applied.

## 4. Discussion

Results show that PM alone or in combination with Mg fertilizer (PM + Mg) increased both the pH and nutrient contents of the soil. The increase in soil nutrient content with PM was adduced to the fact that PM is a natural and effective source of plant nutrients [21, 22]. This was also consistent with the analysis recorded for PM in this experiment showing that it contains these nutrients. PM also has liming effect [23]. NPK alone or in combination with Mg fertilizer (NPK + Mg) increased N, P, K, and Mg contents of the soil.

Mg fertilizer alone increased N, P, K, Ca, and Mg contents of the soil significantly compared with the control. Soil analysis prior to the start of the experiment showed that the soil was deficient of Mg. It has commonly been reported that sandy soils are frequently deficient of Mg [24], whereas fine textured soils (clay) generally contain adequate Mg as Mg is located in clay minerals and associated with cation exchange sites on clay surfaces. The increase in N concentration as a result of Mg fertilizer could be adduced to the fact that Mg seems to enhance N availability in soil and utilization by crops [25–27].

Compared with other treatments, NPK + Mg significantly increased soil N, P, and K concentrations. This was due to high quantities of these elements in NPK fertilizer compared with poultry manure. PM + Mg increased OM, Ca, Mg, and pH compared with NPK fertilizer because the organic materials in PM decomposed to release OM and other nutrients. The ability of PM to increase pH could be due to the presence of base cations contained in the PM [28]. It had been reported that such cations are released upon microbial decarboxylation [29].

PM increased the growth and yield of turmeric in this study compared with the control. This was due to high concentration of nutrients in PM and also due to its low C: N ratio [7.35], which, in turn, would increase OM decomposition and subsequent nutrient release. [10] also reported that turmeric responded well to PM application. The increase in the growth and yield of turmeric as a result of the application of NPK fertilizer was due to the fact that the soil of the experimental site lacked essential nutrients responsible for growth and yield of turmeric [30]. [9] reported response of turmeric to NPK 15-15-15. This response was due to nitrogen in NPK fertilizer promoting leaf growth and required to form proteins and chlorophyll, while P contributes to root development, energy transfer reactions, and cell division and multiplication and K contributes to stem development, cell division, formation and translocation of carbohydrates, and mainly tuber/rhizome development in roots.

Results show significant or higher yields of turmeric where Mg fertilizer is added with PM (PM + Mg) or NPK (NPK + Mg) compared with PM or NPK alone. These results were expected because Mg is known to be a constituent of chlorophyll accounting for about 2.7% of the chlorophyll molecule. It is also a cofactor, an enzyme activator in reaction associated with ATP formation, phosphorylation reaction, and subsequent transfer of phosphate [7]. Therefore, Mg is involved in simultaneously controlling processes responsible for photosynthesis, assimilate production, and partitioning among plant parts [31]. Mg supply was also reported to enhance N uptake by plants [32, 33]. Mg application has also been reported to increase the translocation of assimilate from source to sink organs which increases root growth [5, 34]. The increase in root growth as a result of Mg fertilizer application may aid in the absorption of N and other nutrients in the soil, thereby enhancing growth and rhizome yield of turmeric. [12] reported an increase in rhizome yield of turmeric as a result of MgSO_4_ when applied as a foliar spray.

NPK increased growth and rhizome yield of turmeric compared with PM. Although PM had been reported to increase growth and yield of crops [21], the elemental quantity of N, P, and K in NPK fertilizer was higher than that of PM, which may explain the yield differences between NPK and PM. Turmeric is a high nutrient demanding crop; in this regard, [35] obtained good yields of turmeric rhizomes with the combination of 375, 175, and 237.5 kg of N, P_2_O_5_, and K_2_O, respectively.

NPK + Mg increased yield of turmeric compared with NPK alone, likely due to the complementary effect of NPK and MgO fertilizers. [36] reported that secondary nutrients also play important roles in producing higher yields of turmeric. Also, [37] recommended high application rates of secondary nutrients, i.e., Mg at 22 kg ha^−1^ and Sulphur at 44 kg ha^−1^, and a recommended dose of NPK fertilizer for improved yield of turmeric.

Mg fertilizer (applied alone or in combination with PM or NPK fertilizer) increased mineral and vitamin contents of turmeric rhizomes. This can be related to improved soil chemical properties associated with Mg treatments which, in turn, may have resulted in increased metabolic activity. Mg fertilizer also increased N absorbed by the turmeric plants and resulting in an increase in the number of leaves and photosynthetic activity and enhancing physiological processes leading to higher assimilate production which also resulted in an increase in mineral composition in the rhizomes.

In this experiment, PM alone or applied with Mg fertilizer (PM + Mg) increased Na, K, Mg, Ca, Fe, and vitamins contents of turmeric rhizome compared with NPK fertilizer alone or in combination with Mg fertilizer (NPK + Mg). This might be due to differences in the chemical composition of PM compared to NPK fertilizer and its positive effect on soil ecology and plant metabolism [38]. NPK fertilizer contains only N, P, and K, whereas PM contains both macro- and micronutrients. It therefore seems reasonable that the amount and type of mineral nutrients present in the soil will dictate the quantity and quality of nutrients absorbed by the plant. For example, plants grown under organic agricultural conditions are reported to have higher micronutrient contents than conventionally grown plants [39]. Furthermore, because some chemical reactions in cells involve micronutrients, either directly or indirectly [40] could explain why PM exhibited higher vitamin C content compared with NPK fertilizer [38]. The increased mineral and vitamin contents of turmeric rhizomes as a result of application of NPK + Mg and PM + Mg compared with NPK or PM alone can be adduced to increase soil chemical properties as a result of the Mg fertilizer addition which led to greater metabolic activities and hence higher minerals and vitamins in the NPK + Mg and PM + Mg plots compared with their sole forms.

## 5. Conclusion

This study shows that PM, NPK, and Mg fertilizers alone or combinations increased soil chemical properties, growth, yield, and mineral and vitamin contents of turmeric rhizomes compared with the control. For this experiment, NPK + Mg increased growth and yield of turmeric compared with other treatments. The NPK + Mg treatment increased the yield of turmeric compared with NPK alone, likely due to the complementary effect of NPK and Mg fertilizers. The PM + Mg fertilizer treatment significantly improved mineral and vitamins contents compared with the other treatments. Therefore, for those that desire turmeric rhizomes for their nutritive value, PM + Mg is recommended. For those that want quantity, NPK + Mg is recommended.

## Figures and Tables

**Table 1:** Initial soil characteristics before experimentation.

Property	Value
Sand (%)	68.2
Silt (%)	16.3
Clay (%)	15.5
Textural class	Sandy loam
pH (water)	5.71
Soil organic matter (%)	2.56
Total N (%)	0.18
Available P (mg kg^−1^)	9.50
Exchangeable K (cmol kg^−1^)	0.21
Exchangeable Ca (cmol kg^−1^)	1.23
Exchangeable Mg (cmol kg^−1^)	0.31

**Table 2:** Chemical analysis of poultry manure and chemical fertilizers used.

Property	Poultry manure	NPK fertilizer	MgO fertilizer
pH (water)	6.67		
Ash (%)	13.1		
Organic C (%)	21.1		
Total N (%)	2.8	15	
C: N ratio	7.35		
P (%)	1.41	15	
K (%)	1.86	15	
Ca (%)	0.81		
Mg (%)	0.59		29.41
Na (%)	0.29		
Cu (%)	0.35		
Mn (%)	0.24		
S (%)	0.28		
Zn (%)	0.25		

**Table 3:** Effect of Mg fertilizer, PM, NPK fertilizer, and their combinations on soil chemical properties.

Treatment	pH (water)		Organic matter (%)		Total N (%)		Available P (mg kg^−1^)		Exchangeable K (cmol kg^−1^)		Exchangeable Ca (cmol kg^−1^)		Exchangeable Mg (cmol kg^−1^)	
	2017	2018	2017	2018	2017	2018	2017	2018	2017	2018	2017	2018	2017	2018
Control	5.71b	5.70b	2.52b	2.50b	0.17f	0.16f	9.2f	9.1f	0.19e	0.18e	1.19c	1.20c	0.30d	0.29d
PM	5.95ab	5.99ab	3.15a	3.20a	0.21d	0.22d	16.4d	17.1d	0.26c	0.27c	2.10a	2.21a	0.43c	0.45c
NPK 15-15-15	5.53b	5.51b	2.51b	2.52b	0.24bc	0.25bc	21.6ab	22.4ab	0.28ab	0.29ab	1.20b	1.20b	0.29d	0.29d
MgO fertilizer	5.96ab	5.98ab	2.52b	2.53b	0.19e	0.18e	10.8e	10.0e	0.20de	0.20de	1.26b	1.21b	0.46c	0.48c
PM + MgO	6.10a	6.30a	3.18a	3.20a	0.23c	0.24c	17.3cd	17.8cd	0.27bc	0.29bc	2.15a	2.26a	0.75a	0.79a
NPK + MgO	5.70b	5.71b	2.53b	2.50b	0.26a	0.27a	22.1a	22.6a	0.29a	0.30a	1.24b	1.26b	0.55b	0.56b

Values followed by similar letters under the same column are not significantly different at p = 0.05 according to Duncan's multiple range test; poultry manure = PM.

**Table 4:** Effect of Mg fertilizer, PM, NPK fertilizer, and their combinations on performance of turmeric.

Treatment	Plant height (cm)		Number of leaves		Number of tillers		Number of rhizomes		Fresh rhizome weight (t ha^−1^)	
	2017	2018	2017	2018	2017	2018	2017	2018	2017	2018
Control	51.2d	50.3d	9.3e	9.1e	3.1e	3.1e	9.1e	9.0e	19.4e	19.2e
PM	63.8b	64.1b	12.1c	12.7c	4.4c	4.6c	14.1c	14.3c	25.2c	25.8c
NPK 15-15-15	69.7ab	69.2ab	13.6b	13.9b	4.8b	4.7b	15.2b	15.1b	28.4b	28.1b
MgO fertilizer	54.7cd	54.9c	10.8d	10.8d	3.3de	3.1de	10.3d	10.1d	21.7d	21.1d
PM + MgO	67.4ab	68.9ab	13.1ab	14.1ab	4.9b	5.1b	15.7b	16.0b	27.1b	27.9b
NPK + MgO	73.2a	73.9a	14.3a	14.8a	5.3a	5.6a	16.4a	16.7a	31.6a	30.9a

Values followed by similar letters under the same column are not significantly different at p = 0.05 according to Duncan's multiple range test; poultry manure = PM.

**Table 5:** Effect of Mg fertilizer, PM, NPK fertilizer, and their combinations on mineral and vitamin contents of turmeric rhizome (means of 2017 & 2018 pooled).

	Na (%)	K (%)	Mg (%)	Ca (%)	Fe (%)	Vitamin C (%)	Vitamin A (%)
Control	142.6f	101.7f	221.2e	81.6e	7.2f	1.2f	4.5f
Poultry manure (PM)	256.3c	125.8d	323.3c	136.6ab	17.2ab	2.5ab	13.6b
NPK 15-15-15	201.7d	130.1ab	285.7d	92.4d	14.7d	1.8d	9.5d
MgO fertilizer	192.6e	110.5e	368.3b	100.1cd	12.7e	1.5e	7.3e
PM + MgO	527.8a	129.3cd	402.1a	140.4a	18.1a	2.8a	15.6a
NPK + MgO	321.6b	137.7a	286.3d	96.7cd	15.6cd	2.0c	10.6cd

Values followed by similar letters under the same column are not significantly different at p = 0.05 according to Duncan's multiple range test.

## Data Availability

The data used to support the findings of this study are included within the article.
